# Robotic lower-lip mucosal graft ureteroplasty for ureteral stenosis longer than 2 cm: initial experience of thirteen patients

**DOI:** 10.3389/fsurg.2024.1504867

**Published:** 2024-12-06

**Authors:** Zhaolin Zhang, Xin Zeng, Yuting Wu, Gengqing Wu, Zhihua He, Guoxi Zhang, Xiaofeng Zou, Yuanhu Yuan, Hui Xu

**Affiliations:** ^1^ Department of Urology, First Affiliated Hospital of Gannan Medical University, Ganzhou, Jiangxi, China; ^2^ First Clinical Medical College, Gannan Medical University, Ganzhou, Jiangxi, China

**Keywords:** lower-lip mucosa, reconstructive surgery, robotic ureteroplasty, ureteral stenosis, oral mucosal graft

## Abstract

**Objectives:**

To present our initial experience of robotic ureteroplasty with lower-lip mucosal graft (LLMG) for treating ureteral stenosis longer than 2 cm and evaluate its feasibility and efficacy.

**Materials and methods:**

A total of thirteen patients with ureteral stenosis who underwent robotic ureteroplasty with LLMG were retrospectively analyzed. After identification and dissection of the ureteral stenosis segment, the segment was incised longitudinally. Then, the LLMG was harvested according to the characteristics of stenosis and sutured with the ureter in onlay fashion. All procedures were completed successfully.

**Result:**

The median stenosis length was 3.5 cm (ranged: 3.0–4.5 cm). The mean length and width of the LLMG were 3.81 ± 0.60 cm and 1.27 ± 0.26 cm, respectively. The mean operative time and anastomosis time were 212.31 ± 23.06 min and 36.54 ± 6.58 min, respectively. The double-J stent was removed at 8 weeks postoperatively in all patients. Three patients (23.1%) suffered postoperative complications during the follow-up period (range, 6–18 months), including fever, urinary tract infection and stenosis recurrence. The success rate was 92.3% (12/13).

**Conclusion:**

Robotic ureteroplasty with LLMG is a safe and feasible technique for treating ureteral stenosis.

## 1 Introduction

The ureter can be well protected by surrounding tissue in the retroperitoneal space and is not susceptible to injury. Iatrogenic injury is the most common causative factor of ureteral injury (1), which may result in ureteral stenosis or atresia, hydronephrosis, and even progressive impairment of renal function.

Short stenosis of the proximal and middle ureter can be managed by endoscopic procedures, pyeloureteroplasty, or end-to-end ureteroureterostomy (1). However, the management of long ureteral stenosis (>2 cm) is a challenge for urologists. Although bowel interposition and renal autotransplantation have been applied for long ureteral stenosis, bowel and vascular complications are not uncommon and cannot be ignored (2, 3). Oral mucosal graft (OMG) is well-suited for the urinary tract, and several studies have reported the application of oral mucosal grafts in the treatment of ureteral strictures (4–6). However, to our knowledge, no study has reported the application of lower-lip mucosal graft (LLMG) in ureteroplasty. Based on our experience of urethroplasty with LLMG and the application of the da Vinci Xi surgical system, we performed robotic ureteroplasty with LLMG for proximal and middle ureteral stenosis. We presented our initial experience as follows.

## 2 Materials and methods

### 2.1 Patients

All medical records of patients with proximal and middle ureteral stenosis who underwent robotic ureteroplasty with LLMG in our hospital between November 2022 and January 2024 were retrospectively reviewed. Inclusion criteria: patients with proximal and/or middle ureteral stenosis or atresia who underwent robotic ureteroplasty with LLMG and whose lesion length was longer than 2 cm, which was unsuitable for ureteroureterostomy or pyeloplasty. The exclusion criteria were as follows: (a) combined with urinary carcinoma; (b) open reconstructive surgery; and (c) combined with oral disease. Finally, thirteen patients were enrolled. The surgeries were performed by senior surgeons with extensive experience in urologic reconstruction and expertise in robotic manipulations.

Preoperatively, intravenous urography, retrograde pyelography, computed tomography urography, or magnetic resonance urography were performed to evaluate ureteral stenosis and hydronephrosis. Urinalysis and midstream urine culture were performed, and appropriate antibiotics were administered as necessary. Preoperative demographic characteristics including sex, age, body mass index, preoperative symptoms, surgical side, hydronephrosis, and parameters related to ureteral stenosis were obtained from the medical records.

Ethical approval for the study was obtained from the Ethical Committee of the First Affiliated Hospital of Gannan Medical University (Number: 2023032709), and the study was conducted in accordance with the Declaration of Helsinki (as revised in 2013). Written informed consent was obtained from all participants included in the study.

### 2.2 Surgical techniques

**Figure 1:**
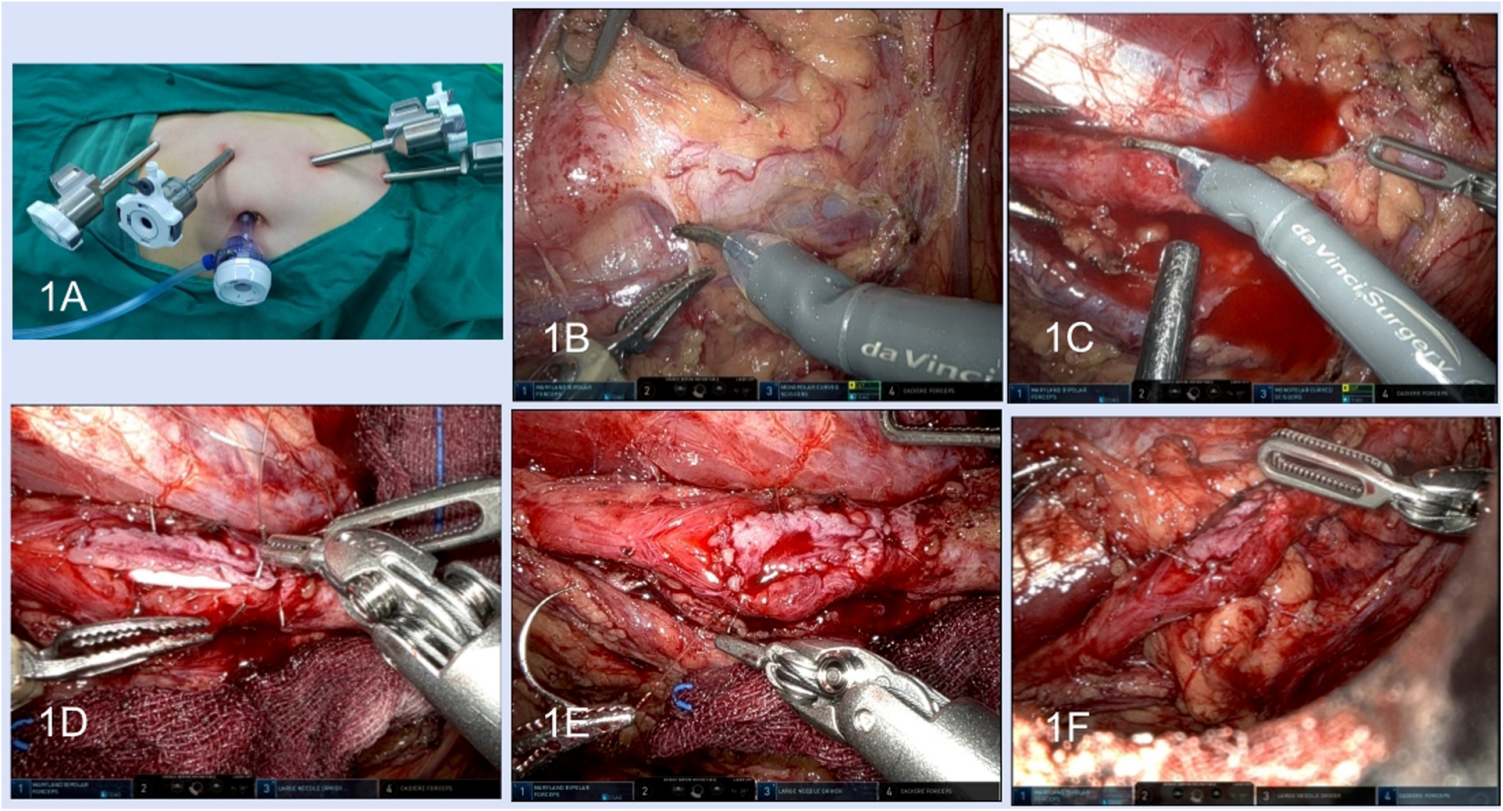
Robotic lower-lip mucosal graft ureteroplasty. **(A)** Surgical trocar placement; **(B)** dissection of the ureter. **(C)** Incision of the stenosis segment. **(D)** Anastomosis of the graft and the ureter wall. **(E)** The graft was sutured in onlay fashion. **(F)** Pedicled omentum wrapped around the anastomosis.

All alternative therapeutic schedules were elaborated to patients, and informed consent was obtained preoperatively. The patient was placed in the supine position or modified supine lithotomy position with the affected side elevated at 45°–70° after general anaesthesia and orotracheal intubation. For the robotic technique, three 8 mm trocars were distributed along the midclavicular line, another 8 mm trocar was placed along the midclavicular line or the anterior axillary line, and an assisted 12 mm trocar was inserted near the umbilicus (Figure 1A).

**Figure 2:**
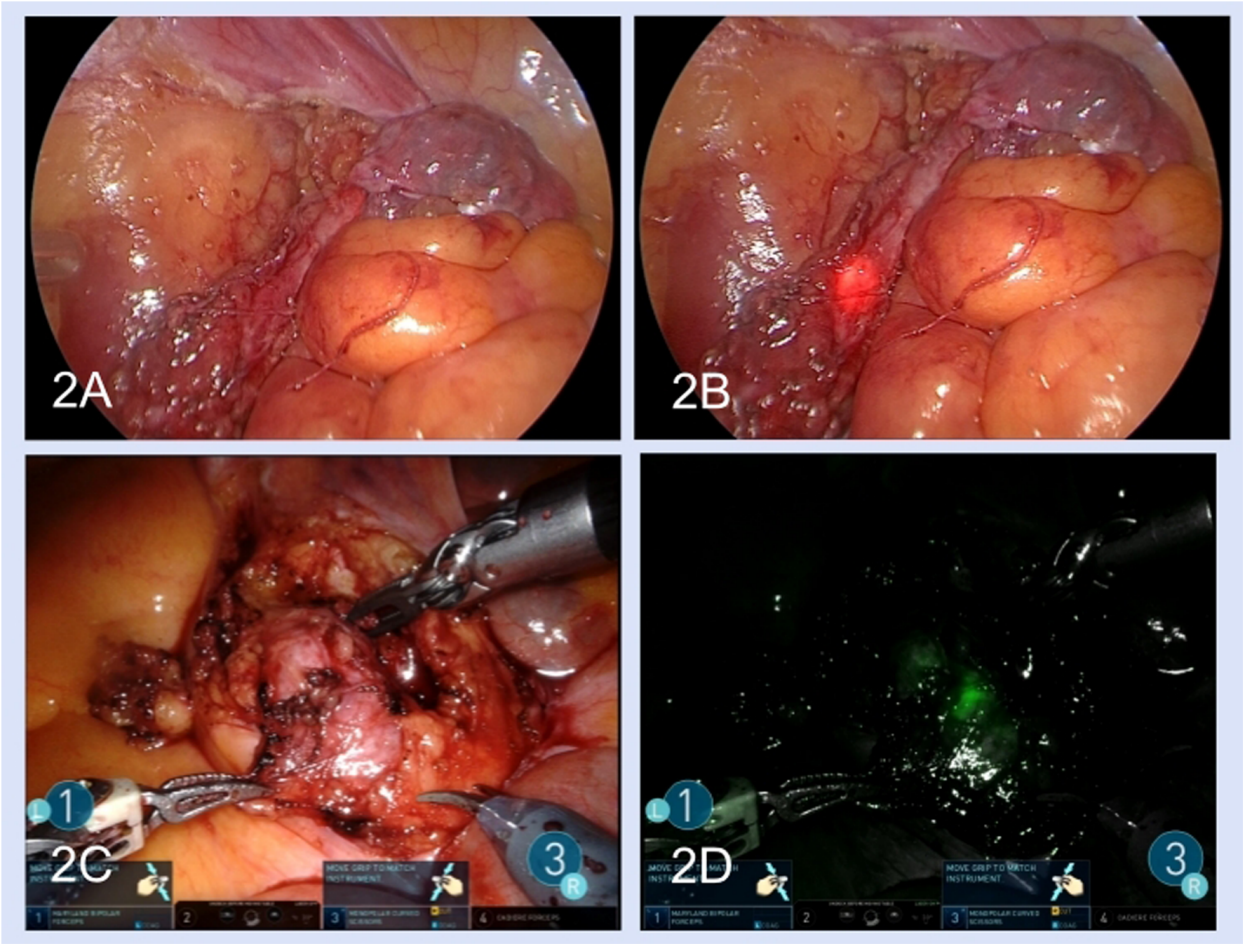
Methods of seeking the stenosis segment: double endoscopic combination **(A,B)** and intravascular indocyanine green **(C,D)**. **(A)** The ureter was seen under the laparoscopic view. **(B)** The luminous tip of the ureteroscope was seen under the laparoscopic view. **(C)** The ureter stenosis segment in laparoscopic light. **(D)** Use of intravascular indocyanine green fluorescence.

After the lateral peritoneum was incised along the Toldt line and the retroperitoneal space was exposed, the ureter was identified based on anatomical markers including the iliac artery, reproductive vessels, or inferior renal pole. Dilatation of the proximal ureter and a thickened scar indicated the location of ureteral stenosis (Figure 1B). Intraoperative ureteroscopy was performed for some complex patients, and the luminous tip of the ureteroscope was located at the distal end of the stenosis, which could be viewed under laparoscopy to identify the stenosis segment (Figures 2A,B). For robotic procedures, intravascular indocyanine green (ICG) was used, if necessary, to facilitate distinguishing the target ureteral segment (Figures 2C,D).

After the hard and cicatricial tissue around the ureter was resected, a longitudinal incision of ureteral stenosis at the ventral side was made with scissors (Figure 1C). The scar tissues on the ureteral wall were excised until the pink healthy ureteral mucosa was exposed at both ends of the ureteral stenosis. The ureteral polyps in the ureter lumen need to be completely excised. The required graft area was marked according to the length and width of the ureteric defect, and 20% shrinkage of the graft size during growth needed to be considered.

**Figure 3:**
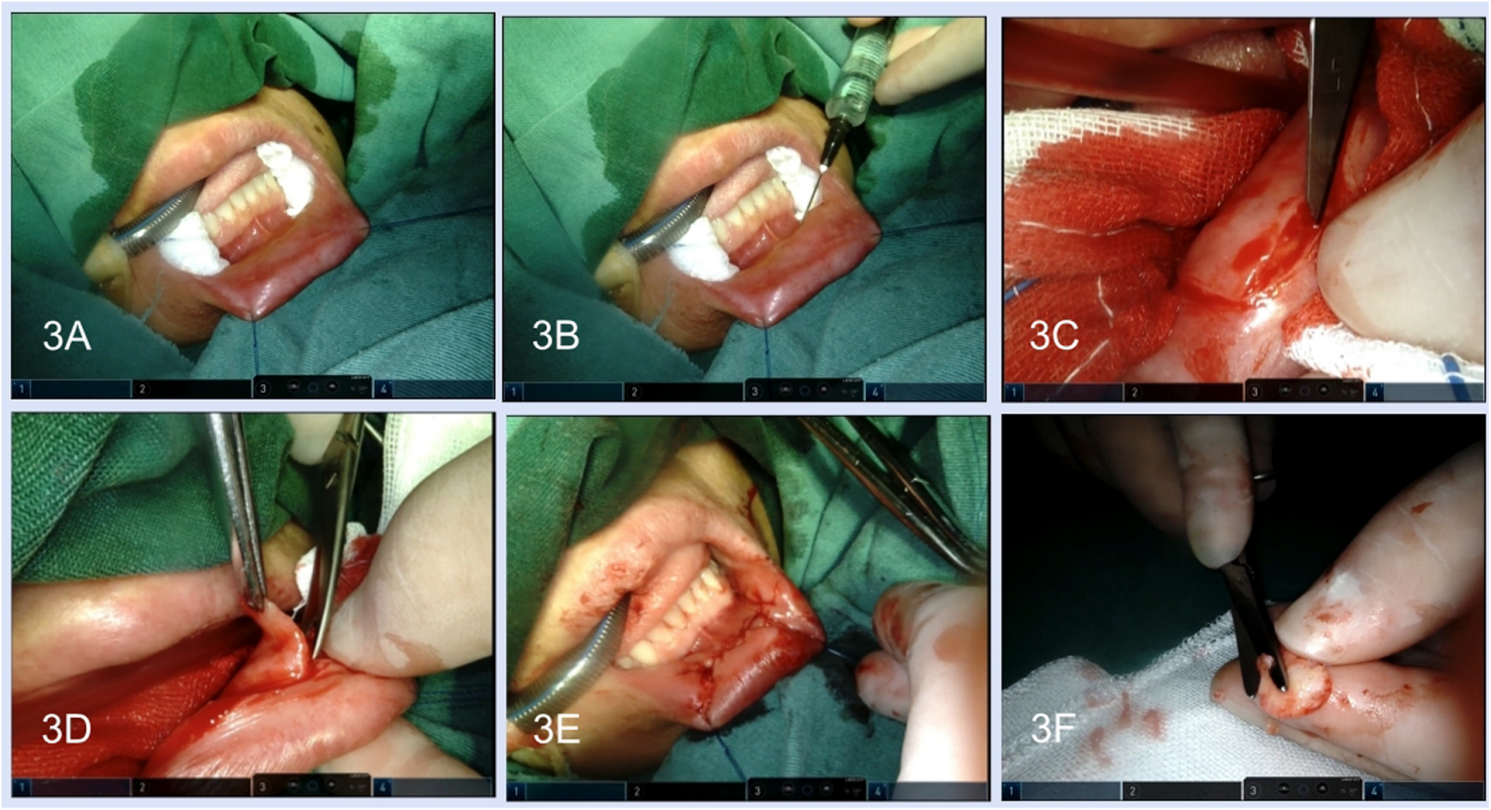
Harvest of the lower-lip mucosal graft. **(A)** Exposure of the lower lip. **(B)** The lidocaine and diluted epinephrine were injected into the submucosal layer. **(C)** Incision was performed along the margin of the graft. **(D)** The graft was freed by scissors. **(E)** Closure of the graft harvest site. **(F)** Removal of the submucosal tissue.

After lidocaine and diluted epinephrine (1:100,000) were injected into the submucosal layer of the lower lip, adequate LLMG was harvested, and then the submucosal tissue of LLMG was removed. The donor site was closed with 5-0 absorbable sutures in an interrupted or running fashion and the incision was compressed with a gelatine sponge until orotracheal intubation was removed (Figures 3A–F).

A 7 F double J stent was inserted across the ureteral defect. The acquired graft was placed into the abdominal cavity and covered the ureteral defect with the epithelium facing the ureteral lumen. Anastomosis was performed between the lateral edge of the graft and the ureteral margin with 4–0 or 5–0 absorbable sutures in a running fashion (Figures 1D,E). The adjacent well-vascularized pedicled omentum was harvested to wrap around the anastomosis (Figure 1F). A 20 Fr drainage tube was inserted and all incisions were closed.

An enhanced recovery program after surgery was applied for postoperative patients. Ambulation was started 1 day after the surgery, and the patient began with a liquid diet, gradually changing to a general diet if tolerated. The drainage tube was removed if the daily volume was ≤20 ml/d. The double J stent was removed at 8 weeks after surgery. All patients were recommended for further consultation at 3, 6, and 12 months postoperatively and then annually after that. The protocols included physical examination, laboratory examination, and imaging studies. Ultrasonography, intravenous urography, computed tomography or magnetic resonance were strongly recommended. Ureteroscopy was also recommended. Surgical success was defined as improvement of hydronephrosis, relief of symptoms, and no recurrent stenosis. Intraoperative and postoperative complications were evaluated according to the Satava (7) and the Clavien-Dindo grade system (8), respectively.

## 3 Results

The demographic characteristics and preoperative data are summarized in Table 1. The etiologies of ureteral stenosis included ureteral Holmium laser lithotripsy, percutaneous nephrolithotomy, open ureterolithotomy and laparoscopic uretero-ureterostomy. A total of seven male and six female patients met the criteria with a mean age of 45.9 ± 13.6 years. Proximal ureteral stenosis was observed in ten patients, and middle ureteral stenosis was detected in three patients.

**Table 1:** Demographic characteristics and baseline data of patients.

Variables	Value
Total number, (*n*)	13
Age (years), mean ± SD	45.9 ± 13.6
Sex, *n* (%)	
Male	7 (53.8%)
Female	6 (46.2%)
Body mass index (Kg/m2), mean ± SD	23.17 ± 4.13
Operative side, *n* (%)	
Left	6 (46.2%)
Right	7 (53.8%)
Preoperative symptoms, *n* (%)	
Flank or/and abdominal pain	11 (84.6%)
No symptoms	2 (15.4%)
Etiology of ureteral stenosis	
Ureteral Holmium laser lithotripsy	9 (69.2%)
Percutaneous nephrolithotomy	2 (15.4%)
Open ureterolithotomy	1 (7.7%)
Laparoscopic uretero-ureterostomy	1 (7.7%)
Ureteral lesion location, *n* (%)	
Proximal ureter	10 (76.9%)
Middle ureter	3 (23.1%)
Hydronephrosis	
Mild	2 (15.4%)
Moderate	5 (38.4%)
Gross	6 (46.2%)

SD, standard deviation.

All surgical procedures were completed without conversion to open surgery or other reconstructive surgeries. No intraoperative complications were observed. The median stenosis length was 3.5 cm (ranged, 3.0–4.5 cm). The mean length and width of the LLMG were 3.81 cm and 1.27 cm, respectively. The mean operative time was 212.31 ± 23.06 min, and the mean anastomosis procedure of the ureter and LLMG was 36.54 ± 6.58 min. The median blood loss was 35 ml (range, 20–60 ml), and none of the patients needed blood transfusion. The median postoperative hospitalization duration was 7 days (range, 5–9 days). All patients underwent removal of the double-J stent at 8 weeks after the operation. The clinical outcomes are presented in Table 2.

**Table 2:** Clinical outcomes of patients.

Variables	Value
Total number, (n)	13
Length of stenosis (cm), median (range)	3.5 (3.0–4.5)
Width of stenosis (cm), mean (range)	1.13 (1.0–1.2)
Length of graft (cm), mean (range)	3.81 (3.0–5.0)
Width of graft (cm), mean (range)	1.27 (1.0–1.5)
Operative time (min), mean ± SD	212.31 ± 23.06
Time of anastomosis procedure (min), mean ± SD	36.54 ± 6.58
Blood loss (ml), median (range)	35 (20–60)
Drainage tube removal time (days), median (range)	4 (3–7)
Postoperative hospitalization (days), median (range)	7 (5–9)
Total complications, Clavien grade classification, *n* (%)	3 (23.1%)
Fever (> 38°C) (G I)	1 (7.7%)
Urinary tract infection (G II)	1 (7.7%)
Stenosis recurrence (G III)	1 (7.7%)
Follow-up time (months), median (range)	12 (6–18)
Success rate, *n* (%)	12 (92.3%)

SD, standard deviation; G, grade.

A total of three patients suffered postoperative complications. One patient suffered from fever (Grade I) and was treated with antipyretics. Urinary tract infection requiring antibiotics (Grade II) was observed in one patient. Complications at the lower lip donor site, including prolonged discomfort, pain, and numbness, were not observed in all patients.

**Figure 4:**
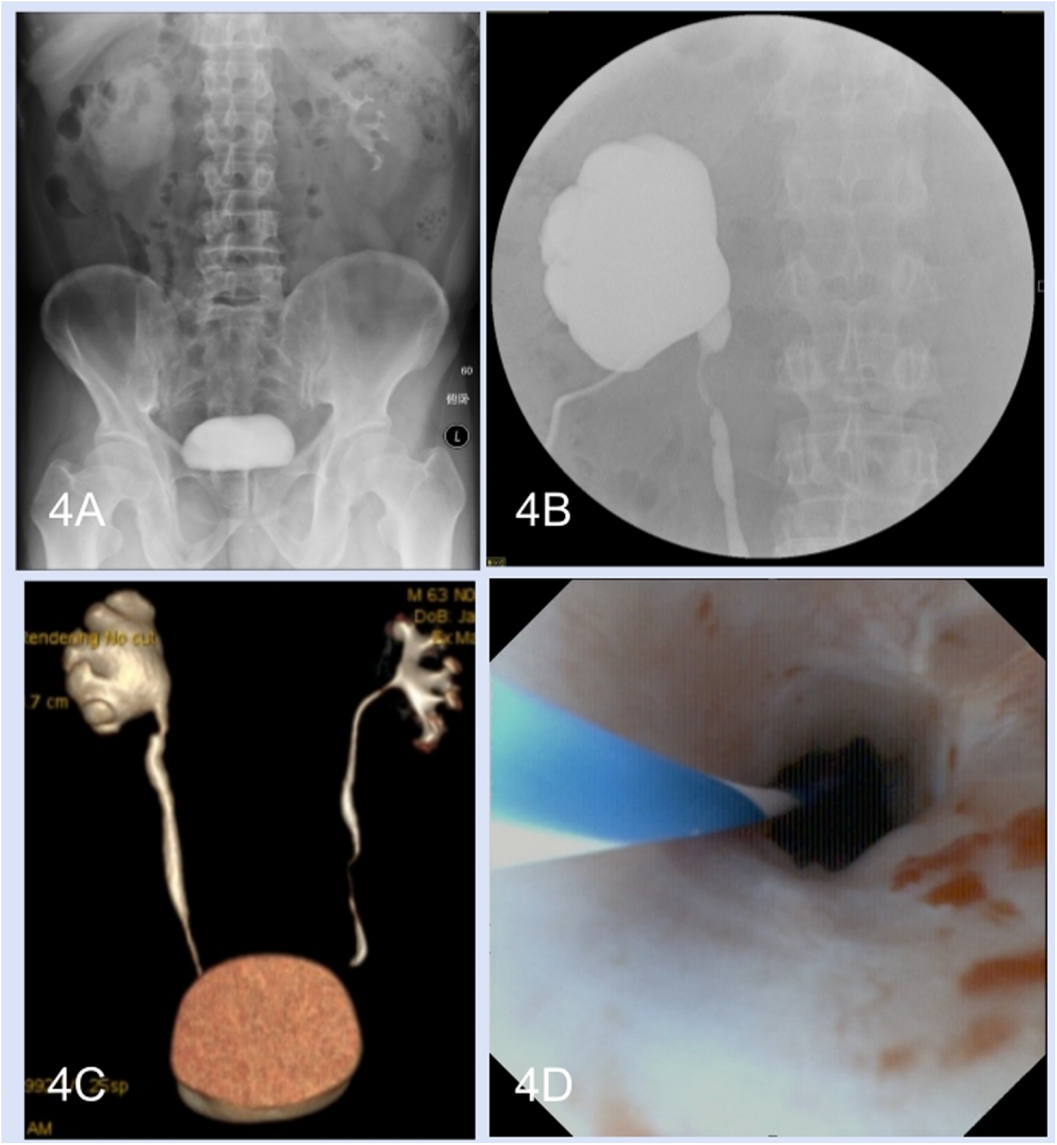
Perioperative radiological examinations and postoperative ureteroscopy. **(A)** Preoperative intravenous urography. **(B)** Anterograde pyelography. **(C)** Postoperative computed tomography urography. **(D)** Postoperative ureteroscopy.

During the follow-up period (range, 6–18 months), all thirteen patients underwent ultrasonography and intravenous urography. Furthermore, computed tomography urography was performed in ten patients and another three patients underwent magnetic resonance urography. Hydronephrosis improvement was observed in 12 (92.3%) patients, and the radiological results indicated good ureteral patency (Figures 4A–D). Preoperative flank or abdominal pain was relieved. Pre- and postoperative ^99^mTc-mercaptoacetyltriglycine renography were performed in seven patients. The preoperative and postoperative glomerular filtration rates of the operative side kidney were 19.57 ± 4.43 ml/min and 26.57 ± 4.92 ml/min, respectively. Seven patients underwent ureteroscopy. One patient experienced renal colic and fever 8 weeks after removal of the double-J stent, after which nephrostomy tube insertion and ureteroscopy were performed. Anastomotic edema and short stenosis (Grade III) were observed, and a 7 F double-J stent was inserted again for 3 months; however, the patient failed to have the double-J stent removed again and was treated with a chronic double-J stent.

## 4 Discussion

Iatrogenic factors are the most common etiology of ureteral injury (9). Ureteral injury may lead to ureteral stenosis, atresia, and ureteral fistula. Endoscopic surgeries and ureteroureterostomy are suitable for short ureteral stenosis. Long-segment stenosis remains an enormous challenge for urologists. Repeated replacement of the ureteral stent and permanent nephrostomy are compromise treatments; however, these treatments may result in poor quality of life and recurrent urinary tract infections. Ureteral reconstructive surgery is still considered the gold standard treatment for long-segmental ureteral stenosis (10). Bowel interposition (appendix, ileum), the bladder flap technique, OMG ureteroplasty, and renal auto-transplantation are optional therapies for long ureteral stenosis.

The OMG has special histological characteristics such as hairless, thick, and non-keratinized epithelium, compatibility with a wet environment, and rich vascularity of the lamina propria (11), and it has been demonstrated to be a good substitute for urethroplasty with good outcomes (12). Based on similar histological characteristics of the urethral mucosa and ureter mucosa, urologists have performed ureteroplasty with OMG for long and complex ureteral stenosis (4–6, 13).

Unlike the bowel interposition and bladder flap, the freed OMG is sutured at the target site of the ureter and acts as part of the ureteral wall. Both the normal ureter distal to the stenosis and the vesicoureteric junction are preserved, so the physiologic peristalsis and anti-reflux functions of the ureter are not affected. Moreover, the OMG does not require the simultaneous transfer of blood supply vessels, however, ileal ureteric replacement requires a segment of the intestine with mesentery, and the harvested bladder flap needs to be attached to the bladder for blood supply.

The donor sites of OMG include the buccal mucosa (inner cheek, lip) and lingual mucosa (tongue) (11). There is no consensus among urologists as to which site is preferred. However, to our knowledge, no study has reported the application of lower-lip mucosal graft (LLMG) in ureteroplasty. All patients in our study had OMG harvested from the lower lip for the following reasons. First, the donor site was easily exposed without the need for a mouth retractor even if the patient was placed in the special position described in our study. Second, orotracheal intubation could meet surgical requirements without the need for nasotracheal intubation. Third, salivatory duct damage can be avoided. Moreover, the postoperative condition of the donor site was easy to check. Kamp et al. compared donor-site complications of the lower lip with those of the inner cheek, and greater long-term morbidity was observed in the lower lip group (14). However, in our study, complications related to donor sites were not observed in any of the patients. Compared to the LLMG, the lingual mucosa could be harvested longer in length, but it was technically harder because a mouth opener was needed and the tongue needed to be pulled out. Complications such as difficulty with fine motor movements, numbness or slurred speech may occur after lingual mucosa harvesting.

The length and width of the graft were measured according to the characteristics of the intraoperative ureteral defect. Importantly, shrinkage of the graft size may have occurred during the growth time when we harvested the graft (15). Some details were emphasized in our study. The length of the LLMG graft was matched that of the defect, and we harvested LLMG with width of 1.0–1.5 cm, which were relatively wider than those of the ureteral defect. The boundary of the graft was marked before submucosal injection to avoid submucosal dilation affecting the true length and width. The bleeding point at the donor site was managed with continuous suturing and compression hemostasis rather than coagulation forceps to reduce thermal damage to blood vessels and nerves.

Comparing to open surgery, robotic ureteral reconstructive surgery has the advantages of less invasion, less bleeding, faster recovery, and shorter postoperative hospitalization (16, 17). Other advantages include image amplification, precise dissection and suturing. Moreover, the robotic surgical system has special superiorities including 3-D stereoscopic vision, multifold magnification of the image, multiple manipulator arms, and delicate operation, which could facilitate intracorporeal suture (18). These features make robotic approaches more suitable for ureteral reconstruction. Zhao et al. first described robot-assisted buccal mucosa graft ureteroplasty, and all four patients were successful during the follow up period (19). Lee et al. reported their multi-institutional experience with robotic ureter reconstruction by using BMG, and surgical success was observed in 87.0% (47/54) of the patients (6). Compared to previous reports, all patients in our study had BMGs harvested from the lower lip, and the success rate was comparable with the results of previous reports (6, 19).

Identification of the ureteral stenosis segment is the key step, and it is sometimes difficult in surgery, especially for patients with previous stenosis-related procedures. The hard and cicatricial tissue surrounding the ureter, which we call the “ureteral armor”, makes it difficult to distinguish the ureteral stenosis segment. Several methods can be used to solve this problem. First, preoperative radiological examinations could indicate the stenosis location; moreover, the gradual decrease in the degree of dilatation of the proximal ureter could help to identifu the stenosis site. Yang et al. applied real-time 3D imaging combined with a surgeon's cognitive fusion during the operation procedure to locate the stenosis and achieved satisfactory effects (5), but special imaging software was needed. Second, the intraoperative combination of ureteroscopy and laparoscopy is a simple and effective method. A modified supine lithotomy position was used, which is beneficial for ureteroscopic insertion. The ureteroscope is retrograded to the distal end of the stenosis, and then the luminous tip of the ureteroscope could be seen from the laparoscope so that we could identify the stenosis site. Second, intraurethral or intravascular ICG fluorescence imaging could facilitate the identification of the stenosis (18). A preexisting ureteral catheter or nephrostomy tube is needed for intraureteral ICG, and the ICG may spill out after the incision of the ureter; thus, the surgical field can be contaminated by ICG, which may influence the subsequent judgement of the ureter condition. However, intravascular ICG can avoid these limitations and was easy to use, and we preferred to use intravascular ICG during surgery.

The reconstructive principles of robotic ureteroplasty are consistent with those of open surgery, including tensionless, watertight, and mucosa-to-mucosa anastomosis, gentle manipulation of the ureter, and sufficient blood supply (17, 20, 21). In accordance with these principles, several technical difficulties must be considered. First, the “ureteral armour” must be completely excised until the ureter appears, and the scar tissues on the ureteral wall were excised until the pink healthy ureteral mucosa was exposed at both ends of the ureteral stenosis. The ureteral polyps in the ureter lumen must be completely excised. These details are important for preventing recurrent stenosis (4, 5, 22). Second, ischemia, infection, and localized adhesion constitute the major factors of failed ureteral reconstruction when the tissue transfer approach was used (23). The blood supply of the freed BMG was partly provided by the ureter. Extensive ureterolysis may disrupt the blood supply of the ureter, so precise positioning of the target segment is particularly important, which could provide maximum protection of the ureteral blood supply. Third, the surrounding tissues, such as perinephric fat and omentum, could provide extra blood supply for freed graft (24) which could improve the survival rate of the graft. Pedicled omentum flaps have been increasingly applied in ureteroplasty because of their its special characteristics, including vascular development and remodelling, anti-infection effects, and regeneration of tissue (25). We preferred to use an omentum flap as covering tissue because the harvested area was large enough for tension-free wrapping.

Except for one patient who experienced recurrent stenosis during the follow-up period, none of the remaining patients experienced recurrent stenosis or increased hydronephrosis. The patient underwent surgery at the early stage of the LLMG urteroplasty procedure, predominantly because of insufficient relevant experience. With the accumulation of experience and the continuous improvement in surgical techniques, our total success rate was 92.3%, which was comparable to what has been reported in previous OMG studies (4, 6).

The study summarized our initial experience and several limitations exist. The main limitation was that it was a retrospective study in a single center, and selection bias could not be avoided. Second, the control group was lacking and the total sample size was small. For further study, a multicenter randomized controlled study is recommended. Third, the follow-up periods of some patients were relatively short, and long-term follow-up is needed in future studies.

## 5 Conclusion

Robotic ureteroplasty with LLMG is a safe and feasible technique for treating ureteral stenosis longer than 2 cm.

## Data Availability

The original contributions presented in the study are included in the article/Supplementary Material, further inquiries can be directed to the corresponding author.
